# Natural Products as GPCR-Targeting Antidepressant Candidates: Advances and Opportunities

**DOI:** 10.3390/ph19081275

**Published:** 2026-08-12

**Authors:** Huayan Li, Xiying He, Ting Cao, Jinfeng Huang, Bojun Chen, Lijing Xu, Yanxiao Yang, Gang Li, Lei Xiong

**Affiliations:** 1 The First Clinical Medical College, Yunnan University of Chinese Medicine, Kunming 650500, China; lhy14101128@163.com (H.L.); xyhetian@foxmail.com (X.H.); 15096686927@163.com (T.C.); 15825097280@163.com (J.H.); 19948751868@163.com (L.X.); 18214663485@163.com (Y.Y.); 2 The Second Clinical Medical College, Yunnan University of Chinese Medicine, Kunming 650500, China; chenbojunyn@163.com; 3 College of Basic Medical Sciences, Yunnan University of Chinese Medicine, Kunming 650500, China

**Keywords:** natural products, G protein-coupled receptors, antidepressant drug discovery, depression, medicinal chemistry, serotonin receptors, cannabinoid receptors, neuroinflammation, signaling pathways

## Abstract

Depression is a leading cause of disability worldwide, and currently available antidepressants are limited by delayed therapeutic onset, inadequate efficacy in some patients, and adverse effects. G protein-coupled receptors (GPCRs), the largest family of membrane receptors in the central nervous system, regulate neurotransmission, neuroplasticity, neuroinflammation, stress responses, and reward processing, and are therefore important targets for antidepressant drug development. Natural products are a rich source of structurally diverse bioactive compounds, many of which show antidepressant-like effects through the modulation of GPCR-mediated signaling pathways. In this narrative review, we summarize the roles of major GPCR families implicated in depression and provide an updated overview of natural products that modulate these receptors. We particularly emphasize receptor-specific mechanisms, downstream signaling networks, and the pharmacological actions of representative natural compounds. We also highlight emerging concepts in GPCR biology, including receptor heteromerization, signaling bias, and allosteric modulation, that may create new opportunities for antidepressant discovery. Finally, we discuss current challenges related to target validation, pharmacokinetics, and clinical translation. Collectively, these insights support further investigation of natural product-derived GPCR modulators as potential leads for next-generation antidepressant development.

## 1. Introduction

Major depressive disorder (MDD) is a prevalent and debilitating psychiatric condition characterized by persistent low mood, anhedonia, and reduced motivation. Because of its high prevalence, recurrent course, and association with suicide risk, MDD represents a major global public health burden. Depression affects more than 300 million individuals worldwide, and its disease burden is expected to continue increasing in the coming decades [1,2]. The World Health Organization has projected that depression will become one of the leading causes of global disability by 2030 [3]. Despite extensive investigation, the etiology and pathophysiology of depression remain incompletely understood and are generally considered to involve complex interactions among genetic susceptibility, environmental stressors, and neurobiological dysfunction. Current mechanistic hypotheses implicate monoaminergic dysregulation, hypothalamic–pituitary–adrenal (HPA) axis dysfunction, neuroinflammation, epigenetic alterations, and impaired neuroplasticity [4].

Currently available antidepressants include tricyclic antidepressants (TCAs), selective serotonin reuptake inhibitors (SSRIs), serotonin–norepinephrine reuptake inhibitors (SNRIs), and norepinephrine–dopamine reuptake inhibitors (NDRIs) [5]. Although these agents are effective in some patients, their clinical utility remains limited by delayed therapeutic onset, inadequate response rates, and adverse effects, including insomnia, headache, sexual dysfunction, and increased suicidal ideation [6]. In recent years, emerging antidepressant strategies targeting noncanonical pathways, including ketamine, a noncompetitive N-methyl-D-aspartate receptor (NMDAR) antagonist, and psilocybin, a 5-hydroxytryptamine 2A (5-HT2A) receptor agonist, have shown rapid and sustained antidepressant effects in clinical and preclinical studies. Nevertheless, concerns regarding psychotomimetic effects, abuse liability, and long-term safety remain unresolved [7,8]. Accordingly, safer and more effective antidepressant strategies with multi-target regulatory properties are urgently needed.

G protein-coupled receptors (GPCRs) constitute the largest family of membrane receptors, with more than 800 members identified in the human genome. These receptors recognize diverse extracellular stimuli, including neurotransmitters, peptides, lipids, and hormones, and transduce intracellular signals primarily through heterotrimeric G proteins [9]. GPCRs contribute to numerous physiological and pathological processes, including neurotransmission, endocrine regulation, immune responses, and synaptic plasticity [10,11]. Many conventional antidepressants exert their therapeutic effects, at least in part, through direct or indirect modulation of GPCR-mediated signaling pathways [12]. Consistent with their broad pharmacological relevance, GPCRs are among the most successful target classes in modern drug discovery. To date, approximately one-third of approved drugs act on GPCRs, including several antidepressants, underscoring the importance of GPCR signaling in depression pharmacotherapy [13,14].

Given the limitations of traditional single-target antidepressants, natural products have emerged as promising sources of neuropsychiatric drug candidates because of their structural diversity and multi-target pharmacological activities, and generally favorable safety profiles [15,16]. As an example, hypericin, a major active ingredient in St. John’s wort extract, shows marked antidepressant activity through multiple mechanisms, accompanied by a favorable side-effect profile [17]. Increasing evidence indicates that numerous natural compounds exert antidepressant-like effects by modulating GPCRs and downstream signaling networks. However, previous reviews have generally focused either on GPCRs as therapeutic targets in depression or on the broad pharmacological activities of natural products, with limited integration of specific natural compounds, their associated GPCR subtypes, and downstream signaling pathways. Consequently, the mechanistic interpretation and translational challenges associated with natural product-derived GPCR modulators in depression remain to be further clarified.

This review summarizes recent advances in natural products associated with GPCR signaling pathways relevant to depression. Here, the term “antidepressant candidate” refers to natural products that exhibit antidepressant-like effects in preclinical models and are supported by mechanistic evidence implicating their involvement in depression-associated GPCR pathways, although most have not yet been clinically validated as antidepressant therapies. The discussion is organized according to reported GPCR-related mechanisms, current evidence supporting receptor involvement, and limitations of existing studies. Emerging concepts in GPCR pharmacology, including biased signaling, allosteric modulation, receptor heteromerization, and receptor regulation, are also discussed to highlight future perspectives for natural product-derived GPCR-targeting antidepressant strategies.

To identify relevant advances in natural product-derived GPCR-targeting antidepressant candidates, literature searches were conducted in PubMed, ScienceDirect, and Google Scholar databases for studies published between 2016 and 2026. The search strategy included combinations of the following keywords: “natural products”, “natural compounds”, “active ingredients”, “antidepressant”, “depression”, “major depressive disorder”, “G protein-coupled receptor”, and specific GPCR subtype names. Furthermore, the methodological considerations and reporting of this narrative review were informed by relevant methodological principles described in the Joanna Briggs Institute (JBI) Manual for Evidence Synthesis [18].

## 2. Overview of GPCRs: Structure, Classification, and Signal Transduction

### 2.1. Structure and Classification of GPCRs

GPCRs are a large family of transmembrane proteins typically composed of a single polypeptide chain with seven transmembrane α-helices (TM1–TM7). These helices are connected by three extracellular loops (ECL1–ECL3) and three intracellular loops (ICL1–ICL3). The extracellular N-terminus and ECLs primarily mediate ligand recognition and binding, whereas the intracellular loops facilitate coupling to downstream effector proteins. The intracellular C-terminus also contains multiple phosphorylation sites and interacts with regulatory proteins, including β-arrestins, thereby contributing to receptor desensitization, internalization, and signal regulation [19]. On the basis of sequence homology and pharmacological properties, GPCRs are generally classified into six families. Four major classes are predominantly expressed in humans: Class A, or rhodopsin-like receptors; Class B, or the secretin receptor family; Class C, or metabotropic glutamate receptors; and Class F, or Frizzled/Smoothened receptors [20].

### 2.2. GPCR Signaling

**Figure 1:**
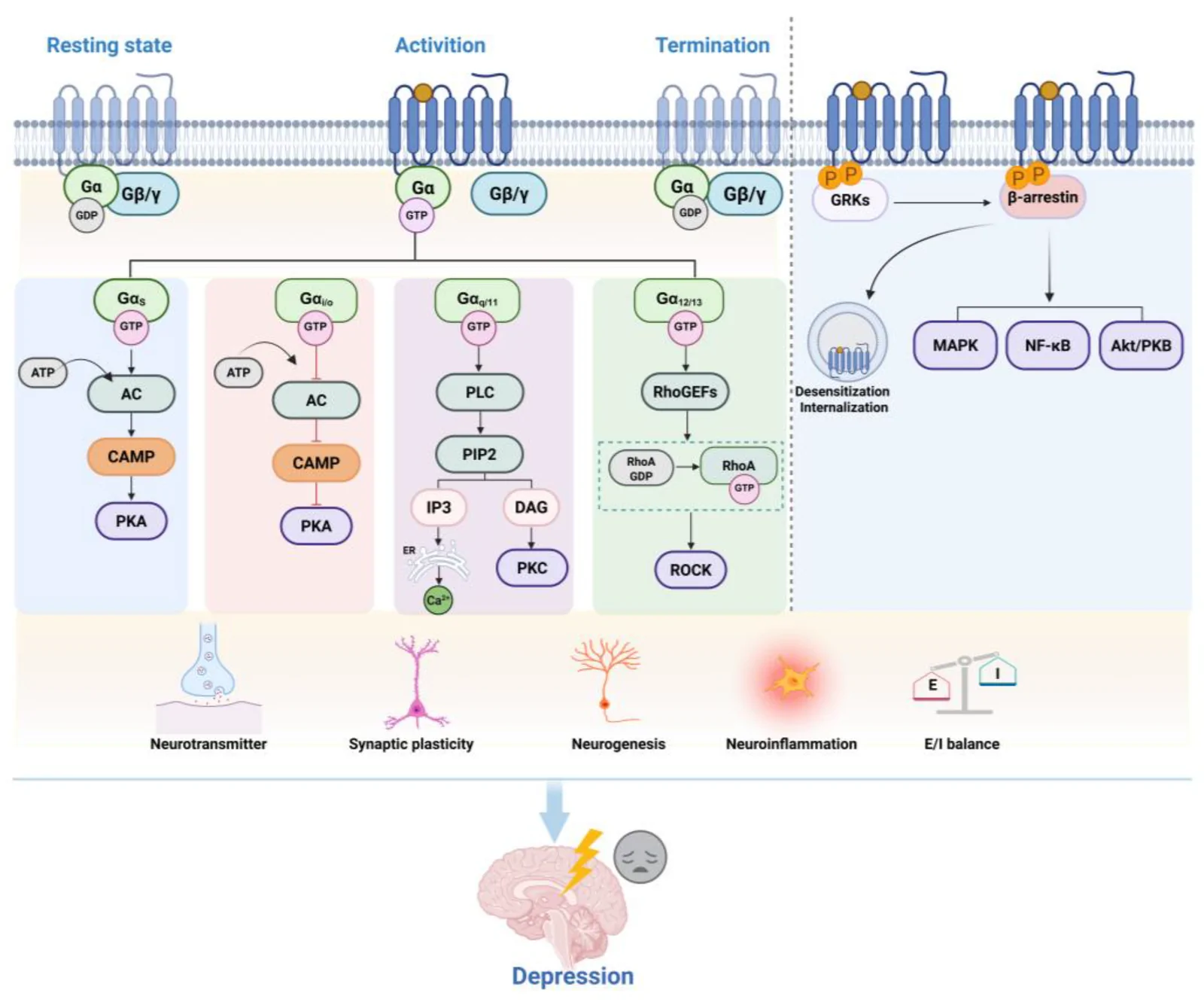
GPCR-mediated signaling pathways involved in depression (created with BioRender.com). GPCRs regulate intracellular signaling through two major pathways: heterotrimeric G protein-dependent signaling and β-arrestin-dependent signaling pathways. Ligand-induced GPCR activation promotes Gα-mediated signaling through four major G protein families (Gαs, Gαi/o, Gαq/11, and Gα12/13), leading to regulation of downstream effectors involved in neurotransmitter homeostasis, synaptic plasticity, neurogenesis, and neuroinflammation. Activated GPCRs can also undergo GRK-mediated phosphorylation and β-arrestin recruitment, triggering receptor desensitization/internalization and downstream signaling cascades, including the MAPK, NF-κB, and Akt/PKB pathways. Natural products may modulate GPCR-associated antidepressant mechanisms at multiple levels, including receptor activation/conformation, G protein or β-arrestin signaling, and downstream intracellular pathways. Black arrows indicate activation or positive regulation, whereas red blunt arrows indicate inhibition or negative regulation. AC, adenylyl cyclase; cAMP, cyclic adenosine monophosphate; DAG, diacylglycerol; GDP, guanosine diphosphate; GPCR, G protein-coupled receptor; GRK, G protein-coupled receptor kinase; GTP, guanosine triphosphate; IP3, inositol 1,4,5-trisphosphate; PIP2, phosphatidylinositol-4,5-bisphosphate; PKA, protein kinase A; PKC, protein kinase C; PLC, phospholipase C; RhoA, Ras homolog family member A; RhoGEF, Rho guanine nucleotide exchange factor; ROCK, Rho-associated coiled-coil-containing kinase.

GPCRs regulate intracellular signaling primarily through two major pathways: the classical heterotrimeric G protein-dependent pathway and the β-arrestin-dependent pathway (Figure 1).

#### 2.2.1. Heterotrimeric G Protein-Dependent Signaling

Heterotrimeric G proteins comprise Gα, Gβ, and Gγ subunits and are located on the cytoplasmic side of the plasma membrane. In the resting state, Gα binds guanosine diphosphate (GDP) and remains associated with the Gβγ dimer. Upon ligand binding, activated GPCRs function as guanine nucleotide exchange factors and promote the exchange of GDP for guanosine triphosphate (GTP) on Gα. The activated Gα–GTP complex subsequently dissociates from Gβγ, and both components independently regulate downstream effectors. Signal transduction is terminated when the intrinsic GTPase activity of Gα hydrolyzes GTP to GDP, allowing reassociation of the heterotrimer [21]. On the basis of structural and functional characteristics, Gα proteins are classified into four major families: Gαs, Gαi/o, Gαq/11, and Gα12/13.

Gαs and Gαi/o: Gαs stimulates adenylyl cyclase (AC), thereby increasing intracellular cyclic adenosine monophosphate (cAMP) levels and activating protein kinase A (PKA). Activated PKA then promotes phosphorylation of cAMP response element-binding protein (CREB), forming the canonical Gαs–cAMP–PKA–CREB signaling axis. This pathway is involved in neurogenesis, synaptic plasticity, and neurotrophic factor expression [22,23,24]. By contrast, Gαi/o inhibits AC activity, reducing cAMP production and suppressing PKA signaling. Through this mechanism, Gαi/o negatively regulates neuronal excitability and neurotransmitter release [25,26]. Dysregulation of the Gαs/Gαi/o signaling balance has been linked to the pathophysiology of depression and other mood disorders.

Gαq/11: Gαq/11 activates phospholipase C (PLC), which catalyzes the hydrolysis of phosphatidylinositol-4,5-bisphosphate (PIP2) into inositol 1,4,5-trisphosphate (IP3) and diacylglycerol (DAG) [27]. IP3 induces Ca^2+^ release from the endoplasmic reticulum, and intracellular Ca^2+^ signaling contributes to neuronal development, neurotransmitter release, and synaptic plasticity [28]. Disrupted Ca^2+^ homeostasis has been implicated in depression and neurodegenerative disorders, including Alzheimer’s disease [29,30,31]. DAG activates protein kinase C (PKC), which regulates synaptic transmission through the phosphorylation of ion channels and neurotransmitter transporters [32]. PKC can also activate downstream pathways, including MAPK/ERK and NF-κB, thereby modulating neurogenesis and neuroinflammatory responses [33,34].

Gα12/13: Gα12/13 signaling primarily activates Rho guanine nucleotide exchange factors (RhoGEFs), which promote the conversion of RhoA from its inactive GDP-bound form to its active GTP-bound form [35]. Activated RhoA subsequently stimulates Rho-associated coiled-coil-containing kinase (ROCK), a central regulator of cytoskeletal remodeling, cell migration, proliferation, and adhesion [36]. Because the actin cytoskeleton is essential for dendritic spine architecture, the RhoA/ROCK pathway contributes to axonal guidance, dendritic spine maintenance, and synaptic structural plasticity [37]. Excessive ROCK activation also promotes glial activation and inflammatory cytokine release, contributing to neuroinflammation associated with depression and neurodegenerative diseases [38].

#### 2.2.2. β-Arrestin-Dependent Signaling

In addition to canonical G protein-dependent signaling, GPCRs can signal through β-arrestin-dependent pathways. β-Arrestins were initially identified as key mediators of GPCR desensitization. After receptor phosphorylation by G protein-coupled receptor kinases (GRKs), β-arrestins bind to the intracellular domain of GPCRs, preventing further G protein coupling and promoting receptor internalization [39,40]. Beyond their canonical role in signal termination, β-arrestins also function as multifunctional signaling scaffold proteins. Independently of, or in cooperation with, G proteins, β-arrestins recruit multiple downstream signaling molecules, including components of the MAPK, NF-κB, and Akt/PKB pathways [41,42]. These signaling cascades contribute to neuronal survival, synaptic plasticity, and neuroinflammatory regulation, and their dysregulation has been associated with the development and progression of depression [43,44]. Importantly, characterization of G protein-dependent and β-arrestin-dependent signaling has provided a mechanistic framework for GPCR-targeted antidepressant discovery, enabling the screening of natural products based not only on receptor binding but also on pathway-selective signaling profiles, target validation, and the identification of compounds with improved efficacy and safety characteristics.

## 3. GPCRs Associated with Depression

### 3.1. Class A: Rhodopsin Family

The rhodopsin family is the largest and most pharmacologically important GPCR class and contains the greatest number of clinically validated drug targets. Rhodopsin-like receptors recognize diverse endogenous ligands, including monoamine neurotransmitters, neuropeptides, purines, prostaglandins, and cannabinoids [20]. Many currently available antidepressants exert their therapeutic effects by directly or indirectly modulating members of this receptor family, particularly monoaminergic receptors such as 5-hydroxytryptamine 1A (5-HT1A) receptors and α2-adrenoceptors [45,46].

#### 3.1.1. 5-HT Receptors

Serotonin, or 5-hydroxytryptamine (5-HT), regulates a broad range of central and peripheral physiological processes, including mood, cognition, sleep, and gastrointestinal function. To date, seven 5-HT receptor families, 5-HT1–5-HT7, have been identified. Except for the ionotropic 5-HT3 receptor, all 5-HT receptors belong to the GPCR superfamily. Among these receptors, 5-HT1 and 5-HT5 primarily couple to Gαi/o proteins, 5-HT2 couples to Gαq/11 proteins, and 5-HT4, 5-HT6, and 5-HT7 couple to Gαs proteins. Among these subtypes, the 5-HT1 and 5-HT2 receptor families are strongly implicated in depression pathophysiology and remain major targets for antidepressant drug development [47].

The 5-HT1 receptor family is the largest serotonin receptor subgroup and includes five subtypes: 5-HT1A, 5-HT1B, 5-HT1D, 5-HT1E, and 5-HT1F. These receptors predominantly couple to Gαi/o proteins and regulate neuronal excitability and neurotransmitter release through modulation of intracellular cAMP signaling and ion channels [48].

The 5-HT1A receptor was the first identified member of the 5-HT1 family [49]. In the central nervous system, 5-HT1A receptors can be broadly classified as presynaptic autoreceptors or postsynaptic heteroreceptors according to their localization and function. Presynaptic autoreceptors in the dorsal raphe nucleus inhibit serotonergic neuronal firing and reduce 5-HT release through negative feedback mechanisms; this process is considered one contributor to the delayed therapeutic onset of SSRIs. By contrast, activation of postsynaptic heteroreceptors in brain regions such as the hippocampus and prefrontal cortex may mediate antidepressant-like effects [50,51]. Increased presynaptic 5-HT1A autoreceptor activity, accompanied by impaired postsynaptic receptor function or serotonergic neuronal loss, has been associated with depression [52]. Accordingly, selective inhibition of presynaptic autoreceptors or preferential activation of postsynaptic heteroreceptors has become an important strategy in antidepressant development [53]. Recently, the novel compound TMU4142 was reported to selectively activate postsynaptic 5-HT1A signaling while avoiding autoreceptor-mediated negative feedback, thereby producing rapid antidepressant-like effects [54].

The 5-HT1B receptor is primarily localized at axon terminals, where it functions as both an autoreceptor and a heteroreceptor regulating neurotransmitter release. 5-HT1B receptors also participate in multiple forms of synaptic plasticity, including long-term potentiation (LTP) in hippocampal–cortical circuits and long-term depression (LTD) at corticostriatal synapses [55,56]. Beyond its involvement in depression-related phenotypes, the 5-HT1B receptor has been identified as a potential mediator of rapid antidepressant-like effects induced by psilocybin and ketamine [57].

The 5-HT2 receptor family comprises three subtypes: 5-HT2A, 5-HT2B, and 5-HT2C. These receptors contribute to emotional regulation and the pathogenesis of psychiatric disorders.

The 5-HT2A receptor is one of the most widely distributed excitatory serotonin receptors in the central nervous system and is predominantly expressed in glutamatergic pyramidal neurons and GABAergic interneurons in the cerebral cortex [58]. This receptor preferentially couples to Gαq/11 proteins and activates phospholipase C (PLC) and phospholipase A2 (PLA2) signaling pathways. PLC hydrolyzes membrane phospholipids to generate IP3 and DAG, leading to intracellular Ca^2+^ release, whereas PLA2 promotes arachidonic acid production. Together, these pathways regulate synaptic transmission and ion-channel activity [59,60]. Because of its role in cortical excitability and synaptic integration, the 5-HT2A receptor is both the principal target of classical psychedelics such as psilocybin and a major antagonistic target of atypical antipsychotics, including risperidone and olanzapine. This dual pharmacological profile highlights its role in mood regulation, cognition, and neuropsychiatric disorders [61,62].

The 5-HT2C receptor is widely expressed in brain regions associated with emotion and reward processing. Like the 5-HT2A receptor, it primarily signals through Gαq/11-mediated pathways. Activation of the 5-HT2C receptor modulates dopaminergic and glutamatergic neurotransmission within cortical and striatal circuits and promotes synaptic plasticity and dendritic growth through multiple downstream effectors [63]. Recent evidence further suggests that 5-HT2C receptors regulate the interaction between neuronal nitric oxide synthase (nNOS) and the carboxy-terminal PDZ ligand of nNOS (CAPON) through Ca^2+^-dependent signaling, thereby influencing γ-aminobutyric acid (GABA) release and contributing to excitation–inhibition balance. Disruption of this process may contribute to depression-related behaviors [29].

Unlike 5-HT2A and 5-HT2C receptors, the 5-HT2B receptor is predominantly expressed in peripheral tissues and is sparsely distributed in the central nervous system, mainly in regions such as the septum and dorsal hypothalamus [64]. Despite this relatively restricted distribution, increasing evidence indicates that 5-HT2B signaling may contribute to antidepressant responses. Pharmacological inhibition or genetic knockdown of 5-HT2B receptors abolishes fluoxetine-induced ERK1/2 activation, suggesting that this receptor may be an important component of SSRI-mediated antidepressant signaling [65].

Given the central role of 5-HT receptor signaling in depression, these receptors have become major targets for natural product-based antidepressant discovery. Several compounds discussed in Section 4, including hirsuteine, psilocybin, curcumin derivatives, and atractylenolide I, exert antidepressant-like effects through modulation of 5-HT receptor-mediated pathways.

#### 3.1.2. Dopamine Receptors

Dopamine (DA) is a major catecholaminergic neurotransmitter that regulates multiple physiological processes, including emotion, reward, motivation, cognition, and motor activity. Dopamine receptors are widely expressed throughout the central nervous system, among which D1 and D2 receptors are the most abundant subtypes [66]. On the basis of structural and pharmacological characteristics, dopamine receptors are classified into two major families: D1-like receptors (D1 and D5) and D2-like receptors (D2, D3, and D4). D1-like receptors mainly couple to Gαs/olf proteins and activate cAMP-dependent signaling, whereas D2-like receptors preferentially engage Gαi/o-mediated pathways and inhibit AC activity [67,68]. Through regulation of neuronal excitability, synaptic plasticity, and reward-related neural circuits, dopamine receptor signaling plays an essential role in emotional processing and depression-associated behaviors [69].

Dopaminergic dysfunction has been particularly implicated in anhedonia, a core symptom of depression characterized by reduced reward sensitivity, motivation, and pleasure. Altered dopamine transmission within mesolimbic circuits, especially involving the ventral tegmental area (VTA) and nucleus accumbens (NAc), contributes to impaired reward processing and depressive phenotypes [70]. Therefore, modulation of dopamine-related GPCR pathways represents an important complementary strategy for antidepressant discovery, particularly for addressing reward and motivational deficits that are insufficiently targeted by conventional monoaminergic therapies.

Recent studies have highlighted the importance of GPCR heteromerization in depression-related signaling. In particular, D1–D2 receptor heterodimers expressed in reward-associated brain regions such as the NAc exhibit signaling properties distinct from those of individual dopamine receptors. Unlike D1 or D2 monomers, which primarily couple to Gαs/olf or Gαi/o proteins, respectively, D1–D2 heterodimers preferentially activate Gαq/11-dependent signaling. This signaling configuration is associated with PLC activation, intracellular Ca^2+^ signaling, and downstream CaMKII activation, which may influence BDNF expression and neuronal plasticity within the NAc and VTA [71]. Altered expression of D1–D2 heterodimers has been observed in the striatum of patients with depression, and dysregulated signaling mediated by these receptor complexes has been implicated in depressive- and anxiety-like behaviors [12]. However, direct evidence linking natural products to dopamine receptor heteromer modulation remains limited, and whether natural compounds can regulate dopamine receptor interactions represents an important area for future investigation.

#### 3.1.3. Opioid Receptors

Opioid receptors are widely distributed throughout the central nervous system and have essential roles in the regulation of mood, reward processing, and pain perception. The classical opioid receptor family comprises the μ-opioid (MOP) receptor, κ-opioid (KOP) receptor, and δ-opioid (DOP) receptor [72]. These receptors predominantly couple to Gαi/o proteins and regulate neuronal excitability, neurotransmitter release, and synaptic plasticity through modulation of intracellular signaling pathways [73]. Accumulating evidence indicates that the opioid system contributes to core depressive symptoms, particularly anhedonia, negative affect, and cognitive dysfunction.

MOP receptor dysfunction has been reported in patients with depression and is associated with impaired reward processing and emotional regulation [74]. In the VTA, the MOP receptor is predominantly expressed on GABAergic interneurons. Activation of the MOP receptor inhibits local GABAergic transmission, thereby disinhibiting dopaminergic neurons and promoting dopamine release within reward-related circuits. This mechanism contributes to the reversal of anhedonia and motivational deficits [75,76]. MOP receptor signaling has also been implicated in fear memory extinction and the upregulation of BDNF expression [77,78], further supporting its role in alleviating negative emotional states.

In contrast to the MOP receptor, the KOP receptor system is generally considered an endogenous anti-reward pathway. Under chronic stress conditions, elevated dynorphin levels lead to excessive KOP receptor activation, which suppresses dopamine release in the NAc and disrupts reward-related signaling. Persistent activation of this pathway contributes to aversive emotional states and depression-like behaviors [79]. Given their ability to normalize stress-induced reward dysfunction, KOP receptor antagonists have attracted considerable interest as potential rapid-acting antidepressants with anti-suicidal properties [80].

The role of DOP receptors in emotional regulation has gained increasing attention in recent years. DOP receptor activation within the VTA promotes dopamine release in the NAc, whereas striatal DOP receptor signaling facilitates reward-seeking behavior by modulating cholinergic interneurons and disinhibiting D1-type medium spiny neurons (D1-MSNs) [79,81]. Several preclinical studies have reported antidepressant-like effects of DOP receptor agonists [82,83], highlighting their potential relevance to mood disorders. However, the pro-convulsant effects associated with classical DOP receptor agonists remain a major obstacle to their clinical development [84]. Collectively, MOP, KOP, and DOP receptors contribute to the pathophysiology of depression through distinct but interconnected regulation of reward, stress, and emotion-related neural circuits. These findings provide an important theoretical basis for the development of novel antidepressant agents targeting opioid receptors, particularly those derived from natural products.

#### 3.1.4. Muscarinic Acetylcholine Receptors

Muscarinic acetylcholine receptors (mAChRs) comprise five subtypes, M1–M5, which are widely distributed throughout the central nervous system and have important roles in cognition, emotion, and reward processing. Increasing evidence suggests that mAChRs represent promising therapeutic targets for depression [85]. Based on their G protein-coupling properties, mAChRs can be broadly divided into two functional groups: M1/M3/M5 receptors that preferentially couple to Gαq/11 proteins and M2/M4 receptors that mainly engage Gαi/o signaling pathways [86].

Among these subtypes, M1 and M2 receptors have received the greatest attention in depression research. M1 receptors are predominantly localized on postsynaptic membranes and are involved in excitatory synaptic transmission [87]. Preclinical studies indicate that M1 receptor inhibition contributes to antidepressant responses. For example, the selective M1 receptor antagonist biperiden produces significant antidepressant-like effects in wild-type mice but not in M1 receptor knockout mice, supporting a receptor-dependent mechanism [88]. Similarly, scopolamine, a non-selective muscarinic receptor antagonist, may exert antidepressant-like effects by blocking M1 receptors on somatostatin-positive interneurons in the medial prefrontal cortex (mPFC). This action disinhibits pyramidal glutamatergic neurons, enhances synaptic plasticity, and restores cortical network activity [89].

Unlike M1 receptors, M2 receptors are expressed on both presynaptic and postsynaptic membranes. Presynaptically, they function as autoreceptors that suppress acetylcholine release, whereas postsynaptically they regulate the release of non-cholinergic neurotransmitters as heteroreceptors [90]. Genetic and clinical studies have linked M2 receptor dysfunction to depression susceptibility. Reduced M2 receptor expression and impaired G protein-mediated signaling have been observed in patients with depression [91]. Beyond its effects on M1 receptors, scopolamine also produces antidepressant-like responses by blocking M2 receptors in the mPFC, thereby activating mammalian target of rapamycin complex 1 (mTORC1)-BDNF signaling. Consistently, intracerebroventricular administration of the selective M2 receptor antagonist methoctramine produces comparable antidepressant-like effects [92].

The emerging antidepressant relevance of muscarinic receptor signaling has also stimulated interest in natural compounds targeting these pathways, exemplified by scopolamine and related agents discussed in later sections.

#### 3.1.5. Adrenergic Receptors

Adrenergic receptors are classical GPCRs activated by epinephrine and norepinephrine and are widely distributed throughout brain regions involved in stress responses, arousal, and emotional regulation. These receptors are classified into three major families: α1-, α2-, and β-adrenoceptors. Through differential coupling to Gαq/11, Gαi/o, and Gαs proteins, respectively, adrenergic receptors regulate intracellular signaling pathways involved in neuronal excitability, synaptic plasticity, and stress adaptation.

Among adrenergic receptor subtypes, the α2-adrenoceptor family has attracted particular attention as a therapeutic target for depression. Postmortem studies have demonstrated pathological upregulation of α2A-adrenoceptors in the hippocampus and prefrontal cortex (PFC) of individuals with depression and suicide-related behaviors [93]. Functionally, α2-adrenoceptors located on noradrenergic neurons in the locus coeruleus serve as presynaptic autoreceptors that negatively regulate norepinephrine release. Excessive α2-adrenoceptor activation suppresses norepinephrine release into the synaptic cleft, thereby impairing monoaminergic neurotransmission. Consequently, pharmacological blockade of presynaptic α2-adrenoceptors, as observed with mirtazapine, removes this inhibitory feedback mechanism, increases synaptic norepinephrine levels, and indirectly enhances serotonergic transmission through activation of α1-adrenoceptors on serotonergic neurons, ultimately producing antidepressant-like effects [94].

Although natural product-derived adrenergic receptor modulators remain less extensively characterized, adrenergic signaling represents an important component of stress-related GPCR regulation in depression.

#### 3.1.6. Cannabinoid Receptors

Cannabinoid receptors mainly comprise two subtypes, cannabinoid receptor 1 (CB1) and cannabinoid receptor 2 (CB2). Both receptors preferentially couple to Gαi/o proteins and suppress intracellular cAMP production through inhibition of AC.

The CB1 receptor is among the most abundant GPCRs in the brain and is highly expressed in regions involved in emotional regulation, reward processing, and cognition, including the cortex, basal ganglia, and hippocampus. Evidence from preclinical studies and clinical observations suggests that CB1 signaling is involved in depression-related behaviors and emotional regulation [95,96]. At the subcellular level, CB1 receptors are predominantly localized on presynaptic membranes, where activation by endogenous or exogenous cannabinoids regulates neurotransmitter release and neuronal excitability through modulation of ion channels and downstream signaling pathways [97]. However, direct CB1 receptor agonists remain difficult to translate clinically because of their psychoactive properties, abuse potential, and adverse effects on cognition.

In contrast, CB2 receptors exhibit a more restricted expression pattern in the central nervous system but are closely associated with immune regulation and neuroinflammatory processes. Increasing evidence suggests that CB2 signaling may influence depression-related behaviors through modulation of microglial activation, inflammatory responses, and stress-related neural circuits [98]. Compared with CB1-targeting approaches, CB2-based strategies may provide potential antidepressant benefits with reduced psychoactive and cognitive risks; however, current evidence remains largely limited to preclinical studies.

Consequently, current therapeutic strategies have increasingly focused on indirect enhancement of endocannabinoid signaling or development of receptor-selective/allosteric modulators to preserve beneficial effects while minimizing adverse outcomes.

Notably, several natural products reviewed in Section 4, including cannabidiol, gastrodin, curcumin, and *Schisandra chinensis* lignans, have been reported to modulate cannabinoid receptor signaling, highlighting the translational potential of the endocannabinoid system in antidepressant drug discovery.

### 3.2. Class C: Glutamate Receptor Family

The Class C GPCR family includes metabotropic glutamate (mGlu) receptors, GABAB receptors, calcium-sensing receptors, type 1 taste receptors, and type 2 vomeronasal (V2) pheromone receptors, the latter of which are highly expressed in rodents but absent in humans. Among these receptors, mGlu receptors are most closely associated with the pathophysiology of depression and have emerged as promising therapeutic targets. mGlu receptors are key regulators of neuronal excitability and synaptic plasticity in the central nervous system and comprise eight subtypes (mGlu1–8 receptors) classified into three groups (Groups I–III) according to sequence homology, G protein-coupling properties, and pharmacological characteristics [99].

Group I mGlu receptors include mGlu1 and mGlu5 receptors. These receptors are predominantly localized in the perisynaptic region of postsynaptic membranes and preferentially couple to Gαq/11 proteins. Through intracellular calcium-dependent signaling pathways, Group I mGlu receptors regulate neuronal excitability and synaptic plasticity, processes critically involved in mood regulation [100]. Among this subgroup, the mGlu5 receptor has received particular attention in stress-related psychiatric disorders. Preclinical studies have shown that negative allosteric modulators of the mGlu5 receptor produce significant antidepressant-like effects, highlighting the mGlu5 receptor as a promising target for antidepressant drug development [101].

Group II mGlu receptors consist of mGlu2 and mGlu3 receptors, which are expressed on both presynaptic and postsynaptic membranes. These receptors primarily couple to Gαi/o proteins and suppress cAMP and cyclic guanosine monophosphate (cGMP) signaling through inhibition of AC and guanylyl cyclase (GC), respectively. By regulating glutamatergic neurotransmission and synaptic plasticity, mGlu2/3 receptor signaling contributes to the maintenance of excitatory–inhibitory balance in neural circuits [102]. Importantly, mGlu2/3 receptors antagonists produce rapid antidepressant-like effects in rodent models that are in some respects comparable to those of ketamine, while exhibiting fewer motor and cognitive adverse effects. These findings suggest that Group II mGlu receptors represent promising GPCR targets for rapid-acting antidepressant therapies [103].

Group III mGlu receptors include mGlu4, mGlu6, mGlu7, and mGlu8 receptors. Except for the mGlu6 receptor, which is primarily restricted to the retina, these receptors are broadly distributed throughout the central nervous system [104]. These receptors regulate synaptic transmission primarily by modulating the release of neurotransmitters [105]. Although research on Group III mGlu receptors in depression remains relatively limited, accumulating evidence indicates that mGlu4 and mGlu7 receptors are involved in emotional regulation and may represent underexplored therapeutic targets for antidepressant intervention.

Collectively, accumulating evidence indicates that multiple GPCR families contribute to depression through complementary regulation of monoaminergic neurotransmission, synaptic plasticity, neuroinflammation, stress responsiveness, and reward processing. Among these receptors, serotonergic, cannabinoid, muscarinic, and metabotropic glutamate receptors have emerged as particularly attractive targets for natural product-based antidepressant discovery because of their established roles in mood regulation and their amenability to pharmacological modulation.

Importantly, many natural products exhibit multi-target pharmacological properties and may simultaneously regulate multiple GPCR-associated pathways, providing potential advantages over conventional single-target approaches. The following section summarizes representative natural compounds targeting these GPCR systems and discusses their mechanisms, pharmacological characteristics, and translational potential as antidepressant candidates.

## 4. Natural Products as GPCR-Targeting Antidepressant Candidates

### 4.1. Alkaloids

#### 4.1.1. Hirsuteine

**Figure 2:**
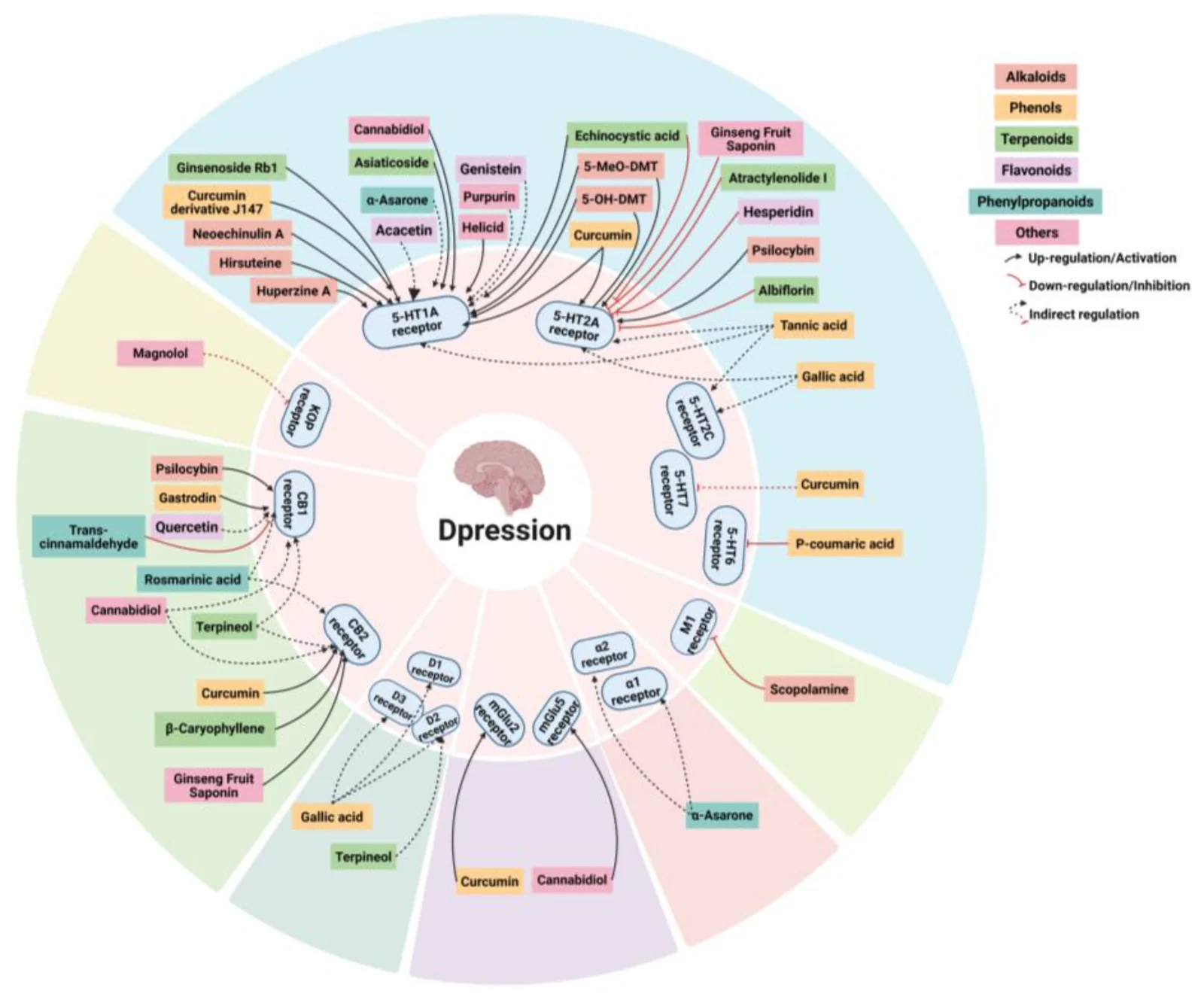
Natural products targeting depression-associated GPCRs (created with BioRender.com). Representative natural products are classified according to their chemical classes and mapped to GPCR families implicated in depression. The connecting lines indicate reported regulatory relationships between natural products and GPCR targets, including upregulation, downregulation, and indirect regulation. Among these GPCR targets, serotonergic receptors are among the most extensively investigated GPCR targets of natural products. The precise molecular mechanisms, receptor selectivity, and signaling bias underlying many natural product–GPCR interactions remain to be further elucidated.

*Uncaria rhynchophylla* has long been used in traditional medicine for central nervous system disorders, including depression and epilepsy. Its major bioactive constituents, particularly alkaloids and flavonoids, exhibit neuropharmacological activities [106]. Hirsuteine, an indole alkaloid isolated from *U. rhynchophylla*, displays agonistic activity toward the 5-HT1A receptor (EC50 = 2.24 μmol/L). In chronic unpredictable mild stress (CUMS) mouse models, hirsuteine alleviated depression-like behaviors and increased hippocampal expression of the 5-HT1A receptor and BDNF (Table 1). In parallel, phosphorylation levels of PKA and CREB were elevated, suggesting activation of the 5-HT1A–PKA–CREB signaling pathway. Mechanistically, molecular docking and site-directed mutagenesis analyses suggested that Asp116 and Asn386 may contribute to the interaction between hirsuteine and the 5-HT1A receptor. Moreover, pretreatment with the selective 5-HT1A receptor antagonist WAY-100635 abolished the antidepressant-like effects of hirsuteine [107,108], supporting the involvement of 5-HT1A receptor signaling in its pharmacological activity (Figure 2).

#### 4.1.2. Neoechinulin A

Neoechinulin A is a prenylated indole alkaloid derived from various fungi, with reported antidepressant-like and neuroprotective activities. Intracerebroventricular administration of neoechinulin A improved lipopolysaccharide (LPS)-induced depression-like behaviors and memory impairment in experimental models. Pharmacological studies showed that these effects were abolished by pretreatment with the selective 5-HT1A receptor antagonist WAY-100635 and the tryptophan hydroxylase inhibitor p-chlorophenylalanine. These findings suggest that the antidepressant-like effects of neoechinulin A are pharmacologically dependent on intact serotonergic neurotransmission and 5-HT1A receptor availability [109]. However, its precise molecular mechanism remains unclear.

#### 4.1.3. Huperzine A

Huperzine A is a tricyclic pyridine alkaloid found in plant species such as *Lycopodium* and *Huperzia serrata*. Because of its acetylcholinesterase inhibitory activity and neuroprotective properties, huperzine A has been widely used in the treatment of Alzheimer’s disease [154]. Recent studies have shown that huperzine A ameliorates depression-like behaviors and cognitive deficits in rat models of post-stroke depression. These effects are accompanied by increased hippocampal expression of the 5-HT1A receptor, BDNF, and phosphorylated CREB. In addition, huperzine A elevates norepinephrine (NE), dopamine (DA), and serotonin (5-HT) levels in the hippocampus and prefrontal cortex [110]. Collectively, these findings suggest that huperzine A may exert antidepressant-like and cognitive protective effects, at least in part, by upregulating 5-HT1A receptor expression and enhancing monoaminergic neurotransmission.

#### 4.1.4. Psilocybin

Psilocybin is a natural tryptamine alkaloid found in mushrooms of the genus *Psilocybe* and is classified as a classic serotonergic hallucinogen. As a non-selective 5-HT2A receptor agonist, psilocybin has shown sustained efficacy in treating treatment-resistant depression in clinical trials [155,156]. In a mouse model of corticosterone-induced depression, psilocybin improved anhedonia, but this effect was absent in 5-HT2A receptor knockout mice [111]. Similarly, a single administration of psilocybin alleviates despair-like behavior in mice with learned helplessness and increases dendritic spine density in cortical pyramidal neurons, with this structural remodeling persisting for at least one month; both effects are blocked after 5-HT2A receptor knockout [112]. Furthermore, recent studies suggest that psilocybin may alleviate behavioral despair and cognitive impairment in a Wistar Kyoto rat model through mechanisms involving CB1 receptor-associated signaling pathways [113]. These findings indicate that its antidepressant-like mechanisms may involve crosstalk among multiple GPCR signaling pathways and may include therapeutic mechanisms that are partly independent of hallucinogenic effects.

Beyond its classical agonist activity at 5-HT2A receptors, emerging evidence suggests that different 5-HT2A receptor ligands may stabilize distinct receptor conformational states, resulting in differential intracellular signaling profiles. Since 5-HT2A receptor activation can engage multiple downstream pathways, including G protein-dependent and β-arrestin-dependent signaling, biased signaling has been proposed as one potential mechanism underlying the dissociation between rapid antidepressant effects and psychedelic-associated adverse responses [157]. Further characterization of psilocybin-associated signaling bias and its relationship with therapeutic and psychedelic effects may provide insights into the development of next-generation 5-HT2A-targeting antidepressants with improved therapeutic windows.

#### 4.1.5. Scopolamine

Scopolamine is a natural tropane alkaloid derived from plants of the Solanaceae family, such as *Scopolia carniolica* or *Datura stramonium*. As a non-selective muscarinic acetylcholine receptor (mAChR) antagonist, it has attracted attention because of its rapid antidepressant effects. Accumulating evidence indicates that M1 receptor signaling in the medial prefrontal cortex (mPFC) contributes to scopolamine-induced antidepressant-like responses. In *Gad1*-Cre mice, selective knockdown of M1 receptors in the mPFC abolished the antidepressant-like effects of scopolamine in the forced swimming test (FST), novelty-suppressed feeding test, and female urine sniffing test [114]. Mechanistically, CUMS reduces expression of the glutamatergic marker vesicular glutamate transporter 1 (VGLUT1) and the GABAergic synaptic protein gephyrin. In this model, a single administration of scopolamine increased c-Fos expression in CaMKIIα-positive pyramidal neurons; however, this effect was absent in M1^f/f^Sst^Cre+^ mice [89]. Further studies showed that scopolamine inhibits M1 receptors on somatostatin (SST)-positive interneurons, thereby reducing inhibitory input onto pyramidal neurons and disinhibiting cortical excitatory circuits. The resulting enhancement of glutamatergic neurotransmission may promote synaptic plasticity-related processes, including increased AMPA receptor function and activation of downstream neurotrophic signaling pathways, which together contribute to its rapid antidepressant-like effects [114]. Beyond its classical receptor antagonism, scopolamine-induced modulation of muscarinic signaling may involve dynamic regulation of receptor function, including adaptive changes in receptor responsiveness and downstream signaling networks. Although GPCR trafficking processes, including receptor internalization and recycling, represent important mechanisms regulating receptor availability and signaling sensitivity, direct evidence demonstrating that scopolamine alters M1 receptor trafficking remains limited.

#### 4.1.6. 5-MeO-DMT and 5-OH-DMT

5-Methoxy-N,N-dimethyltryptamine (5-MeO-DMT) and 5-hydroxy-N,N-dimethyltryptamine (5-OH-DMT, also known as bufotenine) are naturally occurring tryptamine alkaloids primarily derived from *Incilius alvarius*, which are structurally related to serotonin. Both compounds have shown antidepressant-like effects in CUMS models. In mice, a single administration of 5-MeO-DMT reduced immobility time in the FST. However, this effect was abolished by pretreatment with the selective 5-HT2 receptor antagonist ketanserin, suggesting that 5-HT2A receptor signaling contributes substantially to its antidepressant-like activity [111]. Compared with 5-MeO-DMT, 5-OH-DMT retained comparable antidepressant-like efficacy while producing fewer head-twitch responses (HTRs) and greater horizontal locomotor activity (HLA), suggesting a lower hallucinogenic liability and a potentially improved safety profile. Hippocampal knockdown of the 5-HT1A receptor or pharmacological blockade with WAY-100635 attenuated the antidepressant-like effects of 5-OH-DMT. These findings suggest that the behavioral effects of 5-OH-DMT are largely associated with 5-HT1A receptor signaling rather than solely dependent on 5-HT2A receptor activation [115]. This mechanistic distinction may provide structural and pharmacological insights for developing tryptamine-based antidepressant leads with reduced hallucinogenic risk.

### 4.2. Phenols

#### 4.2.1. Curcumin

Curcumin is a diarylheptanoid and the principal polyphenolic constituent of *Curcuma longa* and exhibits diverse pharmacological activities, including anti-inflammatory, antioxidant, and neuroprotective effects. Its antidepressant potential is supported by preclinical and clinical evidence [158].

Accumulating studies indicate that curcumin exerts antidepressant-like effects involving modulation of multiple serotonergic GPCR-associated pathways. In chronic restraint stress (CRS)-induced depression models, curcumin suppresses 5-HT7 receptor-mediated cAMP signaling and ERK1/2 phosphorylation while attenuating NMDAR-associated neurotoxicity, thereby producing neuroprotective and antidepressant-like effects [116]. In ovariectomy-induced depressive models, curcumin upregulates tryptophan hydroxylase 2 (TPH2) and 5-HT1A/2A receptor expression in the limbic system, enhances serotonin synthesis and release, and activates BDNF/ERK signaling, thereby promoting neuroplasticity restoration [117]. The curcumin derivative J147 exhibits high affinity for the 5-HT1A receptor and improves depression-like behaviors through activation of the cAMP/PKA/CREB/BDNF signaling cascade. These effects are abolished by the 5-HT1A receptor antagonists NAD-299 and WAY-100635, supporting the involvement of 5-HT1A receptor-associated signaling in its antidepressant-like activity [120,121].

Beyond serotonergic regulation, curcumin also modulates other GPCR-associated pathways implicated in depression. Curcumin restores mGlu2 receptor/peroxisome proliferator-activated receptor gamma coactivator-1α (PGC-1α) signaling, thereby maintaining glutamatergic homeostasis and mitochondrial function and alleviating corticosterone-induced neurotoxicity and depression-related phenotypes [118]. In Bacillus Calmette–Guérin (BCG)-induced depressive models, curcumin exerts antidepressant-like effects involving CB2 receptor-associated signaling. Pharmacological studies further showed that these effects are attenuated by the CB2 receptor antagonist AM630 and enhanced by the CB2 receptor agonist JWH133 [119].

Collectively, curcumin may exert multimodal antidepressant-like effects through coordinated regulation of GPCR-associated pathways involving serotonergic receptors, metabotropic glutamate receptors, and CB2 receptor signaling.

#### 4.2.2. p-Coumaric Acid

p-Coumaric acid is a natural phenolic acid found in plants such as *Vaccinium bracteatum* Thunb. Previous studies have shown that p-coumaric acid exerts antidepressant-like effects by inhibiting neuroinflammation and upregulating BDNF expression [159]. Recent evidence further suggests that its antidepressant-like activity is associated with GPCR modulation, particularly modulation of the 5-HT6 receptor. In 1321N1 cells stably expressing the human 5-HT6 receptor, p-coumaric acid acted as a functional antagonist (IC50 = 3.65 μM), suppressing Gαs-mediated cAMP accumulation and ERK1/2 phosphorylation. By contrast, in CRS-induced depression models, p-coumaric acid activated ERK/CaMKII and Akt/mTOR/p70S6K/S6 signaling pathways, enhanced synaptic plasticity, and improved depression-like behaviors. This apparent divergence between in vitro inhibitory effects and in vivo activation of plasticity-related signaling may reflect complex circuit-level regulation of 5-HT6 receptors. Specifically, antagonism of 5-HT6 receptors may relieve inhibitory constraints on cortico-hippocampal synaptic plasticity, thereby indirectly enhancing neuronal circuit function. Collectively, current evidence suggests that p-coumaric acid exerts antidepressant-like effects through mechanisms involving functional antagonism of the 5-HT6 receptor, suppression of cAMP signaling, and subsequent activation of neuroplasticity-associated pathways. Nevertheless, the receptor selectivity and precise in vivo regulatory mechanisms of p-coumaric acid require further investigation [122].

#### 4.2.3. Tannic Acid

Tannic acid (TA) is a naturally occurring polyphenol widely distributed in medicinal plants, including *Rhus chinensis* Mill. In LPS-induced depression models, TA exhibits antidepressant-like and neuroprotective effects. Pharmacological studies showed that these effects are abolished by antagonists of 5-HT1A and 5-HT2A/2C receptors, suggesting that serotonergic GPCR signaling contributes to its antidepressant-like activity. In addition, TA reduces monoamine oxidase A (MAO-A) activity and suppresses activation of the Toll-like receptor 4 (TLR4)/NF-κB/IL-1β signaling pathway, thereby attenuating neuroinflammatory responses associated with depression [123]. Collectively, these findings suggest that TA exerts antidepressant-like effects through coordinated modulation of serotonergic neurotransmission and neuroinflammatory pathways.

#### 4.2.4. Gastrodin

Gastrodin, a phenolic glycoside, is the principal bioactive constituent of *Gastrodia elata* Blume, a traditional Chinese medicinal herb used for neurological disorders because of its sedative and hypnotic properties. Recent studies suggest that gastrodin has therapeutic potential in post-stroke depression (PSD) through modulation of CB1-associated signaling. In PSD models, gastrodin increases CB1 receptor expression and enhances downstream PKA signaling, which subsequently suppresses excessive RhoA activation. These effects improve hippocampal synaptic transmission and structural plasticity, including increased dendritic spine density, thereby alleviating depression-like behaviors [124]. These findings suggest that CB1 receptor-associated signaling may contribute to the effects of gastrodin in PSD.

#### 4.2.5. Gallic Acid

Gallic acid is a naturally occurring polyphenolic acid (trihydroxybenzoic acid) widely found in plants such as tea and oak, with reported antidepressant-like activity. Pharmacological studies indicate that gallic acid increases synaptic levels of serotonin and catecholamines, thereby improving depression-like behaviors. Mechanistically, its antidepressant-like effects appear to involve coordinated modulation of multiple GPCR subtypes, including 5-HT2A/2C, dopamine D1, D2, and D3, and adrenergic receptors. These effects appear to be independent of 5-HT1A receptor and β-adrenoceptor signaling, highlighting a specific pharmacological dependence on distinct monoaminergic networks rather than broad receptor activation [125]. The ability of gallic acid to regulate multiple monoaminergic GPCR pathways illustrates the potential value of natural products for targeting complex neurobiological networks underlying depression.

### 4.3. Terpenoids

#### 4.3.1. Atractylenolide I

Atractylenolide I (ATR) is a sesquiterpene lactone isolated from *Atractylodis Macrocephalae Rhizoma* and exhibits anti-inflammatory and neuroprotective activities. In CUMS-induced depression models, ATR ameliorated depression-like behaviors and increased hippocampal levels of DA, NE, and 5-HT. Mechanistically, ATR selectively downregulated 5-HT2A receptor expression without affecting 5-HT2B or 5-HT2C receptor expression. The antidepressant-like effects of ATR were abolished in 5-HT2A receptor knockout mice, suggesting that 5-HT2A receptor signaling is required for its pharmacological activity. Molecular docking analyses predicted potential interactions between ATR and the 5-HT2A receptor, suggesting that ATR may influence 5-HT2A receptor-associated signaling; however, direct receptor-binding and functional validation remain to be established [126].

#### 4.3.2. Echinocystic Acid

Echinocystic acid (EA) is a naturally occurring pentacyclic triterpenoid isolated from *Codonopsis lanceolata*. In reserpine-induced mouse models of pain–depression comorbidity, EA exhibited analgesic and antidepressant-like effects. Mechanistically, EA reversed reserpine-induced downregulation of the 5-HT1A receptor and upregulation of the 5-HT2A receptor, suggesting that alterations in serotonergic receptor expression may contribute to its pharmacological activity. In addition, EA suppressed abnormal expression of excitatory synaptic proteins, including GluN2B, phosphorylated GluA1 (Ser831), Postsynaptic density protein 95 (PSD-95), and CaMKII, thereby regulating excitatory neurotransmission and downstream oxidative stress-related cascades [127]. These findings suggest that EA exerts integrated antidepressant-like and analgesic effects through coordinated regulation of serotonergic signaling and glutamate-dependent synaptic plasticity.

#### 4.3.3. Ginsenoside Rb1

Ginsenoside Rb1 (GRb1) is a triterpene glycoside and one of the major bioactive saponins isolated from *Panax ginseng* C.A. Mey. In LPS-induced depression models, GRb1 alleviated depression-like behaviors. Mechanistic studies showed that GRb1 restored hippocampal 5-HT levels and increased 5-HT1A receptor expression, suggesting involvement of serotonergic GPCR signaling in its antidepressant-like activity. In addition, GRb1 suppressed MAPK/NF-κB-mediated neuroinflammatory responses and ameliorated tryptophan–kynurenine metabolic dysregulation through downregulation of indoleamine 2,3-dioxygenase 1 (IDO1), thereby contributing to antidepressant-like effects [128]. Collectively, these findings suggest that GRb1 exerts multimodal antidepressant-like effects through coordinated regulation of serotonergic neurotransmission, neuroinflammation, and tryptophan metabolism.

#### 4.3.4. Albiflorin

Albiflorin, a glycosylated monoterpene and the principal bioactive constituent of *Paeonia lactiflora*, exhibits antidepressant-like effects in CRS-induced depression rat models. Mechanistically, albiflorin suppresses aberrant activation of the NO/cGMP signaling pathway and reverses stress-induced upregulation of the hippocampal 5-HT2A receptor. In parallel, it attenuates HPA axis hyperactivity and restores levels of monoaminergic neurotransmitters, including 5-HT, DA, and NE. In addition, albiflorin enhances hippocampal BDNF expression, thereby promoting neurogenesis and conferring neuroprotective and antidepressant-like effects [129].

#### 4.3.5. Asiaticoside

Asiaticoside, a characteristic triterpenoid saponin derived from asiatic acid and isolated from *Centella asiatica* (L.) Urb., ameliorates depression-like behaviors in CUMS mice. This antidepressant-like effect is accompanied by attenuation of peripheral inflammation and endocrine dysfunction. Specifically, asiaticoside decreases levels of pro-inflammatory cytokines, including IL-6 and TNF-α, as well as corticotropin-releasing hormone (CRH) and corticosterone (CORT), thereby alleviating systemic inflammatory responses and normalizing HPA axis hyperactivity. Concurrently, asiaticoside elevates peripheral 5-HT levels and upregulates hippocampal 5-HT1A receptor and BDNF expression, suggesting that its antidepressant-like activity is associated with modulation of serotonergic signaling and neurotrophic pathways [130].

#### 4.3.6. β-Caryophyllene

β-Caryophyllene, a bicyclic sesquiterpene widely distributed in plant sources such as clove (*Syzygium aromaticum*) and *Cinnamomum cassia*, exerts antidepressant-like effects primarily through modulation of CB2 receptor signaling. As a selective CB2 receptor agonist, β-caryophyllene reverses stress- or inflammation-induced downregulation of hippocampal CB2 receptor expression. Moreover, it suppresses COX-2-mediated neuroinflammatory responses, enhances BDNF expression, and ameliorates LPS-induced abnormal enhancement of LTD, collectively contributing to its neuroprotective and antidepressant-like properties [131].

#### 4.3.7. Terpineol

Terpineol, a representative monoterpenol natural product widely found in the essential oils of plants such as pine, exhibits antidepressant-like activity mediated through multiple GPCR-related pathways. Its behavioral effects are abolished by dopaminergic receptor antagonists, including haloperidol and sulpiride, the selective CB1 receptor antagonist AM281, and the selective CB2 receptor antagonist AM630, suggesting involvement of dopaminergic and endocannabinoid signaling. In addition, molecular docking analyses have predicted potential interactions between terpineol and CB1, CB2, and D2 receptors, providing theoretical support for direct receptor binding, although further functional validation is required. Collectively, these findings suggest that cannabinoid and dopaminergic receptor-associated signaling pathways may contribute to its antidepressant-like effects [132].

### 4.4. Flavonoids

#### 4.4.1. Acacetin

Acacetin, a naturally occurring apigenin-derived flavonoid widely distributed in plants such as *Agastache rugosa*, exhibits antidepressant-like effects associated primarily with serotonergic signaling. Pharmacological blockade with the selective 5-HT1A receptor antagonist WAY-100635 dose-dependently abolishes the antidepressant-like activity of acacetin, whereas antagonists targeting other 5-HT receptor subtypes, including 5-HT1B, 5-HT2A/2C, and 5-HT3 receptors, do not produce similar effects. Moreover, in vitro studies showed that acacetin potentiates 8-OH-DPAT-induced activation of CHO cells expressing the 5-HT1A receptor, suggesting a positive modulatory effect on 5-HT1A receptor signaling. These findings suggest that acacetin exerts antidepressant-like effects primarily through indirect enhancement of 5-HT1A receptor-mediated serotonergic neurotransmission [133].

#### 4.4.2. Hesperidin

Hesperidin (HSP), a naturally occurring flavanone glycoside abundant in citrus species such as sweet orange, ameliorates depression-like behaviors in CUMS-exposed mice. This behavioral improvement is accompanied by restoration of 5-HT and DA levels, reduction in corticosterone concentrations, and attenuation of oxidative stress and inflammatory responses. Co-administration of the 5-HT2A receptor agonist 2,5-dimethoxy-4-iodoamphetamine hydrochloride abolishes the antidepressant-like effects of HSP, suggesting a role for 5-HT2A receptor signaling in its mechanism of action. Furthermore, molecular docking and molecular dynamics analyses have suggested binding affinity between HSP and the 5-HT2A receptor. Collectively, these findings suggest that HSP exerts antidepressant-like effects through modulation of 5-HT2A receptor-mediated signaling, normalization of HPA axis function, and suppression of neuroinflammatory and oxidative stress pathways [134].

#### 4.4.3. Quercetin

Quercetin, a naturally occurring flavonol widely distributed in plants such as *Sophora japonica* and *Allium cepa*, alleviates CUMS-induced anhedonia and despair-like behaviors. Electrophysiological studies further showed that quercetin reduced the frequency and amplitude of miniature excitatory postsynaptic currents (mEPSCs) in neurons of the lateral habenula (LHb), a brain region involved in depression-related circuitry. These behavioral and synaptic effects are abolished by the CB1 receptor antagonist AM251, suggesting that CB1 receptor signaling within the LHb is involved in the antidepressant-like actions of quercetin [135].

#### 4.4.4. Genistein

Genistein, the major bioactive isoflavone derived from soybean, produces dose-dependent antidepressant-like effects in both the FST and tail suspension test (TST). These behavioral improvements are accompanied by increased brain levels of 5-HT and NE, as well as inhibition of MAO-A activity. Pharmacological studies have shown that the selective 5-HT1A receptor antagonist WAY-100635 abolishes the antidepressant-like effects of genistein, whereas antagonists targeting other 5-HT receptor subtypes, including ritanserin, isamoltane, and ondansetron, do not exert similar effects. Moreover, co-administration of the 5-HT1A receptor agonist 8-OH-DPAT further enhances the behavioral efficacy of genistein. These findings suggest that the antidepressant-like activity of genistein is primarily associated with 5-HT1A receptor-dependent serotonergic mechanisms, in addition to its effects on monoamine metabolism [136].

### 4.5. Phenylpropanoids

#### 4.5.1. α-Asarone

α-Asarone, a phenylpropene and the principal bioactive constituent of *Acorus tatarinowii* Schott, exhibits antidepressant-like activity through modulation of adrenergic and serotonergic neurotransmission. Pharmacological studies have shown that pretreatment with α1-adrenoceptor, α2-adrenoceptor, or 5-HT1A receptor antagonists abolishes its antidepressant-like effects, suggesting involvement of these receptor systems. These findings suggest that α-asarone exerts antidepressant-like activity, at least in part, through an indirect mechanism that requires functional α1/α2-adrenergic and 5-HT1A receptors [137].

#### 4.5.2. Trans-Cinnamaldehyde

Trans-cinnamaldehyde, the major bioactive component of *Cinnamomum cassia*, produces antidepressant-like effects in the FST. Mechanistic investigations have shown that trans-cinnamaldehyde treatment is associated with reduced hippocampal CB1 receptor protein expression, suggesting involvement of endocannabinoid system-related GPCR signaling. These findings suggest that modulation of CB1 receptor-dependent pathways may contribute to the antidepressant-like actions of trans-cinnamaldehyde [138].

#### 4.5.3. Rosmarinic Acid

Rosmarinic acid, a naturally occurring phenylpropanoid widely distributed in *Rosmarinus officinalis* and other medicinal plants, alleviates LPS-induced depression-like behaviors. Its antidepressant-like effects are abolished by pretreatment with either the CB1 receptor antagonist AM281 or the CB2 receptor antagonist AM630, suggesting a role for endocannabinoid signaling. In addition, rosmarinic acid modulates peroxisome proliferator-activated receptor-γ (PPAR-γ)-related pathways, suggesting functional crosstalk between cannabinoid receptor signaling and downstream PPAR-γ-mediated neuroprotective mechanisms. Collectively, these findings suggest that the antidepressant-like and neuroprotective effects of rosmarinic acid are pharmacologically dependent on CB1 and CB2 receptor availability, highlighting crosstalk with downstream PPAR-γ-mediated mechanisms [139].

### 4.6. Other Natural Products

#### 4.6.1. Cannabidiol

Cannabidiol (CBD), a non-psychoactive phytocannabinoid derived from *Cannabis sativa* L., has attracted attention because of its antidepressant potential, particularly the rapid antidepressant-like effects observed in preclinical models. Acute administration of CBD into the ventromedial prefrontal cortex (vmPFC) reduces immobility time in the FST, an effect abolished by pretreatment with either the CB1 receptor antagonist AM251 or the 5-HT1A receptor antagonist WAY-100635, suggesting that both CB1 and 5-HT1A receptors are involved in its antidepressant-like action [140]. Consistent with these findings, the antidepressant-like and analgesic effects of CBD in models of diabetic neuropathy and chronic neuropathic pain-associated depression are attenuated or abolished by pharmacological blockade of CB1, CB2, or 5-HT1A receptors, further supporting the involvement of endocannabinoid and serotonergic signaling pathways [141,144].

At the molecular level, CBD reverses stress-induced upregulation of miR-16 and miR-135 in the vmPFC of CUMS rats and restores 5-HT1A receptor expression. Blockade of the 5-HT1A receptor abolishes these behavioral benefits, supporting the role of serotonergic signaling in mediating CBD-induced antidepressant-like effects [143]. In addition, studies using the Flinders Sensitive Line (FSL) rat model of hereditary depression have shown that CBD-induced antidepressant-like responses are associated with modulation of mGlu5 receptor, ERK1/2, and synaptophysin signaling, independent of changes in endogenous cannabinoid levels [142]. Collectively, current evidence suggests that CBD exerts antidepressant-like effects through coordinated modulation of multiple GPCR-associated signaling pathways, including CB1, CB2, 5-HT1A, and mGlu5 receptors-related mechanisms.

Pharmacologically, the broad effects of CBD are associated with a multifaceted profile extending beyond classical orthosteric activation, particularly involving the allosteric regulation of GPCRs. CBD has been reported to act as a negative allosteric modulator (NAM) of CB1 receptors; by altering the potency and efficacy of orthosteric ligands, it modulates endocannabinoid signaling and may mitigate maladaptive consequences of excessive CB1 receptor activation, such as receptor desensitization or tolerance [160]. This pharmacological profile may distinguish CBD from direct CB1 receptor agonists, which are limited by psychoactive effects, cognitive impairment, and abuse liability. Meanwhile, CB2-related modulation may represent an alternative therapeutic strategy by targeting neuroimmune and inflammatory pathways with reduced psychoactive liabilities. Furthermore, CBD has been reported to interact with opioid receptor systems through allosteric mechanisms, including reported modulation of MOP and DOP receptors, suggesting broader regulatory effects on GPCR pharmacology that warrant further investigation in depression-related disorders [161]. In addition, CBD may enhance 5-HT1A receptor-mediated neurotransmission, which may contribute to its antidepressant-like effects. Collectively, these findings position CBD as a representative natural product with diverse GPCR regulatory properties and support further investigation of its therapeutic potential in depression.

Although numerous preclinical studies have demonstrated antidepressant-like effects of CBD, clinical evidence remains limited. A randomized clinical trial reported that four weeks of CBD administration improved emotional exhaustion and depressive symptoms in frontline healthcare workers. However, additional well-designed clinical studies are required to further evaluate the efficacy, safety, pharmacokinetic properties, and therapeutic potential of CBD in depressive disorders.

#### 4.6.2. Purpurin

Purpurin, a naturally occurring anthraquinone isolated from *Rubia tinctorum* L., exhibits antidepressant-like activity through modulation of serotonergic signaling. Pharmacological studies have shown that the selective 5-HT1A receptor antagonist WAY-100635 abolishes the antidepressant-like effects of purpurin, whereas antagonists targeting other 5-HT receptor subtypes do not produce similar effects. Furthermore, co-administration of the 5-HT1A receptor agonist 8-OH-DPAT enhances its behavioral efficacy. These findings suggest that 5-HT1A receptor-mediated serotonergic neurotransmission is critically required for the antidepressant-like effects of purpurin [145].

#### 4.6.3. Helicid

Helicid, a phenolic glycoside isolated from *Helicia nilagirica* Bedd., exhibits antidepressant-like effects in CUMS rats, as reflected by increased sucrose preference and reduced behavioral despair. These behavioral improvements are accompanied by attenuation of HPA axis hyperactivity and suppression of neuroinflammatory responses. Mechanistically, helicid enhances hippocampal 5-HT1A receptor-associated signaling and subsequently upregulates the ERK/CREB/BDNF pathway, thereby promoting neuroplasticity and contributing to its antidepressant-like efficacy [146].

#### 4.6.4. Magnolol

Magnolol, a neolignan isolated from the bark of *Magnolia officinalis*, has shown antidepressant potential in preclinical studies. In a corticosterone (CORT)-induced mouse model of depression, magnolol selectively suppresses overexpression of dynorphin A (DYN A) in the ventral dentate gyrus of the hippocampus. Given that DYN A is the endogenous ligand of the KOP receptor, its reduction is associated with attenuation of KOP receptor-mediated pathological signaling, thereby alleviating depression-like behaviors [147].

#### 4.6.5. Ginseng Fruit Saponin

Ginseng fruit saponins (GFS), the major bioactive constituents of ginseng fruit, are triterpene saponins that comprise a complex mixture of more than 30 identified ginsenosides—primarily classified into 20(S)-protopanaxadiol and 20(S)-protopanaxatriol categories—and exhibit antidepressant-like activity through modulation of serotonergic signaling. Early studies showed that GFS downregulated platelet 5-HT2A receptor expression and decreased serotonin transporter (SERT) levels in serum and platelets, thereby reducing peripheral 5-HT reuptake and increasing circulating 5-HT concentrations [162]. Similar effects have been observed in models of myocardial infarction (MI)-associated depression, in which GFS treatment increased serum and platelet 5-HT levels while suppressing 5-HT2A receptor expression in both platelets and brain tissue [148]. Further mechanistic studies showed that GFS downregulated cortical 5-HT2A receptor and SERT expression, resulting in reduced serotonin reuptake, enhanced synaptic 5-HT availability, and improvement of depression-like behaviors after MI [149]. Collectively, these findings suggest that regulation of 5-HT2A receptor-dependent serotonergic neurotransmission represents a key mechanism underlying the antidepressant-like effects of GFS.

#### 4.6.6. *Schisandra chinensis* Lignans

*Schisandra chinensis* lignans (SCL), the principal bioactive constituents of *Schisandra chinensis*—including specific compounds such as schisandrin, schisandrol B, schisantherin A, deoxyschisandrin, and γ-schisandrin—have been identified as functional modulators of the CB2 receptor and exhibit antidepressant-like effects in experimental models of depression. Mechanistically, SCL promotes the CB2 receptor/STAT6 signaling pathway, promoting microglial polarization toward the anti-inflammatory M2 phenotype. This shift attenuates neuroinflammation, inhibits neuronal apoptosis, and facilitates neurogenesis [150]. In addition, SCL negatively regulates the PERK–eIF2α pathway through CB2 receptor-dependent mechanisms, reducing calreticulin (CRT) translocation to the neuronal surface and thereby limiting aberrant phagocytic signaling while preserving neuronal homeostasis [151]. SCL also enhances microglial production of BDNF through CB2 receptor-mediated pathway and promotes its delivery to neurons via exosome-mediated communication, thereby improving synaptic plasticity and mitigating neuronal injury [152]. Beyond its central actions, SCL exerts regulatory effects on the gut–brain axis. Engaging CB2 receptor-associated signaling increases FAAH expression and reduces levels of the endocannabinoid anandamide (AEA), thereby alleviating depression-associated intestinal barrier dysfunction while producing anti-inflammatory and anti-apoptotic effects [153]. Taken together, SCL may exert multifaceted antidepressant-like effects primarily through CB2 receptor-mediated signaling. By coordinately regulating microglial activation, neurotrophic support, neuroinflammation, neuronal homeostasis, and gut–brain communication, SCL may represent a promising natural-product lead targeting the endocannabinoid system for depression treatment.

## 5. Conclusions

Depression is a multifactorial neuropsychiatric disorder involving complex interactions among monoaminergic dysfunction, HPA axis dysregulation, neuroinflammation, oxidative stress, and impaired synaptic plasticity [163]. As the largest family of membrane receptors in the central nervous system, GPCRs regulate emotional processing, reward-related behaviors, stress adaptation, and neuroimmune communication. In this narrative review, we summarized recent advances in natural products that target GPCR-related pathways relevant to depression treatment. Current evidence indicates that structurally diverse natural compounds, including alkaloids, terpenoids, flavonoids, polyphenols, and related phytochemicals, exert antidepressant-like effects through modulation of multiple GPCR subfamilies, particularly serotonergic, dopaminergic, opioid, adrenergic, muscarinic acetylcholine, cannabinoid, and metabotropic glutamate receptors.

A prominent feature of these natural products is their ability to regulate interconnected signaling networks rather than single molecular targets. In addition to directly modulating GPCR activity, many compounds influence downstream pathways involved in neurotransmitter homeostasis, neuroinflammation, neurotrophic signaling, mitochondrial function, and synaptic remodeling. Such network-level pharmacological actions may be particularly relevant to depression, which involves dysregulation across multiple molecular, cellular, and circuit-level processes. Compounds such as psilocybin, scopolamine, and several cannabinoid-related molecules have been reported to produce rapid antidepressant-like effects accompanied by enhanced synaptic plasticity and restoration of neural circuit function, suggesting the therapeutic potential of GPCR-directed interventions beyond conventional monoaminergic mechanisms.

The translational relevance of natural products is further supported by clinical experience with several representative compounds. Some natural products have extensive historical human exposure or preliminary clinical evidence, which may provide valuable information for further drug development [155,156]. Curcumin has also shown beneficial effects in patients with depressive disorders [164], whereas huperzine A has been clinically used for cognitive impairment and Alzheimer’s disease [165]. These observations support continued investigation of natural products as potential sources of antidepressant drug leads.

Overall, natural product-derived GPCR modulators represent a promising therapeutic strategy for depression. However, further mechanistic validation and translational studies are required to determine their clinical applicability and to optimize their therapeutic potential.

## 6. Future Perspectives

Despite considerable progress, several challenges remain in translating natural product-derived GPCR modulators into clinically effective antidepressant therapies. Most available evidence is derived from preclinical studies, and the current literature may be influenced by publication bias, as positive antidepressant-like findings are more likely to be reported than negative or inconclusive results. Moreover, commonly used behavioral paradigms, including FST and TST, are valuable for initial screening but cannot fully capture the complex emotional, cognitive, and social dimensions of human depression. In parallel, the pharmacological characterization of many natural products remains incomplete, as evidence for direct receptor engagement and specific GPCR signaling regulation is often limited. Future studies integrating receptor-binding assays, structural analysis, and advanced pharmacological approaches will be important to clarify direct GPCR modulation and improve target validation of natural product-derived candidates.

Emerging concepts in GPCR pharmacology, particularly biased signaling or functional selectivity, provide new opportunities for developing safer antidepressant agents. Natural products, with their structurally diverse scaffolds and multi-target properties, may represent valuable resources for discovering functionally selective GPCR modulators. Biased signaling enables ligands to preferentially engage specific intracellular pathways associated with therapeutic effects while limiting activation of signaling cascades related to undesirable responses [166]. Several GPCR targets discussed in this review, including CB1, μ-opioid, and 5-HT2A receptors, are associated with potential safety concerns such as abuse liability, cognitive alterations, or hallucinogenic effects. Therefore, future investigations should explore whether natural product-derived scaffolds can be optimized to achieve pathway-selective GPCR modulation, thereby improving efficacy while minimizing adverse effects.

Beyond conventional single-receptor targeting, GPCRs can form heteromeric complexes that possess distinct functional and pharmacological properties. GPCR heteromerization, receptor compartmentalization, and spatial organization within cellular microdomains represent emerging mechanisms that may influence antidepressant responses [167]. Although synthetic ligands targeting GPCR heteromers have demonstrated therapeutic potential, the role of natural products in regulating receptor–receptor interactions remains largely unexplored. Determining whether natural compounds can regulate GPCR heteromer formation and signaling properties may provide additional opportunities for precision antidepressant development.

A major obstacle for the development of neuroactive natural products is their suboptimal drug-like properties, including limited blood–brain barrier penetration, poor aqueous solubility, rapid metabolism, and insufficient oral bioavailability. Moreover, safety evaluation, including toxicity assessment and long-term tolerability studies, remains essential for determining their clinical feasibility. Future efforts should therefore focus on improving pharmacokinetic profiles through structural optimization, semi-synthetic modification, prodrug development, and advanced delivery systems. Nanoparticle-based formulations, lipid-based carriers, and other innovative delivery technologies may enhance central nervous system exposure and improve the therapeutic applicability of natural product-derived GPCR modulators [168]. In addition, structure–activity relationship (SAR) studies and computational approaches may facilitate the optimization of natural product scaffolds with improved potency, selectivity, and pharmacokinetic properties.

The development of clinically effective GPCR-targeting antidepressants will require integration of molecular pharmacology, computational approaches, and translational neuroscience. Computational strategies, including virtual screening and molecular modeling, may accelerate the identification of promising natural product scaffolds, while experimental and clinical validation will remain essential for confirming their therapeutic potential. Ultimately, combining mechanistic insights with translational studies may facilitate the development of safer and more effective natural product-derived GPCR modulators.

## Figures and Tables

**Table 1:** Natural Products Taring GPCRs with Antidepressant Potential.

Class	Components	Formula	Modeling Method	Animals or Cell Type	Behavioral Testing Evaluation	Receptors	Evidence Level	Ref.
Alkaloids	Hirsuteine	C_22_H_26_N_2_O_3_	CUMS	C57BL/6 mice	SPT; FST; TST; EPM	5-HT1A	I	[108]
	Neoechinulin A	C_19_H_21_N_3_O_2_	LPS	ddY mice	Y-maze; FST; TST	5-HT1A	II	[109]
	Huperzine A	C_15_H_18_N_2_O	MCAO + CUMS	Sprague–Dawley rats	SPT; FST; MWM; BWT	5-HT1A	III	[110]
	Psilocybin	C_12_H_17_N_2_O_4_P	CORT	C57BL/6 mice	FST; HTR; SPT	5-HT2A	II	[111]
	Psilocybin	C_12_H_17_N_2_O_4_P	LH	C57BL/6 mice	TST; HTR; FS	5-HT2A	II	[112]
	Psilocybin	C_12_H_17_N_2_O_4_P	SIS + POE	Wistar-Kyoto rats	FST; SPT; NOR; OFT; EPM	CB1	III	[113]
	Scopolamine	C_17_H_21_NO_4_	N/A	Transgenic mice (*Gad1*-Cre mice, *Camk2a*-Cre mice) WT C57BL/6 mice	FST; NSFT; FUST	M1	II	[114]
	Scopolamine	C_17_H_21_NO_4_	CUMS	C57BL6 mice M1f/fSstCre+ mice	FST; NSFT; FUST; ST; EPM; LMA	M1	II	[89]
	5-MeO-DMT	C_13_H_18_N_2_O	N/A	C57/BL6 mice	HTR; FST; SPT	5-HT2A	II	[111]
	5-MeO-DMT 5-OH-DMT	C_13_H_18_N_2_O C_13_H_18_N_2_	CUMS	C57BL/6 mice	TST; FST; SPT; OFT; MWM	5-HT1A 5-HT2A	I	[115]
Phenols	Curcumin	C_21_H_20_O_6_	CRS	ICR mice CHO-K1 cells Human astrocytoma 1321N1 cells	FST	5-HT7A	I	[116]
	Curcumin	C_21_H_20_O_6_	Ovariectomy	Wistar rats	FST	5-HT1A 5-HT2A	III	[117]
	Curcumin	C_21_H_20_O_6_	CORT	C57BL/6 mice	OFT; TST	mGlu2	II	[118]
	Curcumin	C_21_H_20_O_6_	BCG	Swiss albino mice	SPT; OFT; TST; ST	CB2	II	[119]
	Curcumin derivative J147	C_18_H_17_F_3_N_2_O_2_	N/A	ICR mice	FST; TST; LMA	5-HT1A	I	[120]
	Curcumin derivative J147	C_18_H_17_F_3_N_2_O_2_	N/A	ICR mice	FST; TST	5-HT1A	II	[121]
	P-coumaric acid	C_9_H_8_O_3_	CRS	C57BL/6 mice 1321N1 cells	FST	5-HT6	I	[122]
	Tannic acid	C_76_H_52_O_46_	LPS	Swiss mice	FST; TST; OFT	5-HT1A 5-HT2A 5-HT2C	II	[123]
	Gastrodin	C_13_H_18_O_7_	MCAO + SRS	C57BL/6 mice	SPT; TST; FST; OFT	CB1	I	[124]
	Gallic acid	C_7_H_6_O_5_	N/A	BALB/c mice	TST; FST; LMA	5-HT2A 5-HT2C D1, D2, D3	II	[125]
Terpenoids	Atractylenolide I	C_15_H_18_O_2_	CUMS	C57BL/6 mice	EPM; OFT; TST; FST	5-HT2A	I	[126]
	Echinocystic acid	C_30_H_48_O_4_	reserpine	C57BL/6 mice	FST; TST; OFT	5-HT1A 5-HT2A	III	[127]
	Ginsenoside Rb1	C_54_H_92_O_23_	LPS	ICR mice	FST; TST	5-HT1A	III	[128]
	Albiflorin	C_23_H_28_O_11_	CRS	Sprague Dawley rats	SPT; OFT; EPM	5-HT2A	III	[129]
	Asiaticoside	C_48_H_78_O_19_	CUMS	C57BL/6 mice	FST; OFT; SPT	5-HT1A	III	[130]
	β-Caryophyllene	C_15_H_24_	CRS	Sprague-Dawley rats	TST; FST	CB2	III	[131]
	Terpineol	C_10_H_18_O	LPS	Swiss mice	TST; ST; OFT	CB1 CB2 D2	II	[132]
Flavonoids	Acacetin	C_16_H_12_O_5_	N/A	C57BL/6J mice CHO cells expressing human 5-HT1A receptors	FST; TST; LMA	5-HT1A	I	[133]
	Hesperidin	C_28_H_34_O_15_	CUMS	Swiss Albino mice	FST; OFT; SPT	5-HT2A	I	[134]
	Quercetin	C_15_H_10_O_7_	CUMS	C57BL/6J mice	TST; FST; OFT; SPT; NSFT	CB1	II	[135]
	Genistein	C_15_H_10_O_5_	N/A	ICR mice	FST; TST	5-HT1A	II	[136]
Phenylpropanoids	α-Asarone	C_12_H_16_O_3_	N/A	ICR mice	TST; LMA; HWT	α1, α2 5-HT1A	II	[137]
	Trans-cinnamaldehyde	C_9_H_8_O	N/A	BALB/c mice	FST	CB1	III	[138]
	Rosmarinic acid	C_18_H_16_O_8_	LPS	Swiss mice	TST; ST; OFT	CB1 CB2	II	[139]
Others	Cannabidiol	C_21_H_30_O_2_	N/A	Wistar rats	FST; OFT	5-HT1A CB1	II	[140]
	Cannabidiol	C_21_H_30_O_2_	Neuropathic pain (chronic constriction injury)	Wistar albino rats	FST	5-HT1A CB1	II	[141]
	Cannabidiol	C_21_H_30_O_2_	Flinders Sensitive Line	FRL and FSL rats	FST; OFT	mGlu5	III	[142]
	Cannabidiol	C_21_H_30_O_2_	CUMS	Sprague Dawley rats	FST; OFT	5-HT1A	II	[143]
	Cannabidiol	C_21_H_30_O_2_	Type 1 diabetes mellitus (T1DM)	Wistar rats	FST; OFT; EPM	5-HT1A CB1 CB2	II	[144]
	Purpurin	C_14_H_8_O_5_	N/A	C57BL/6J mice	FST; TST; LMA	5-HT1A	II	[145]
	Helicid	C_13_H_16_O_7_	CUMS	Sprague-Dawley rats	SPT; OFT; FST	5-HT1A	III	[146]
	Magnolol	C_18_H_18_O_2_	CORT	Oprk1^lox/lox^ mice	FST; TST; SPT; OFT	KOP	II	[147]
	Ginseng Fruit Saponin	N/A	MI + FST	Sprague-Dawley Rats	FST	5-HT2A	III	[148]
	Ginseng Fruit Saponin	N/A	MI	C57BL/6 mice	SPT; FST; TST	5-HT2A	III	[149]
	*Schisandra chinensis* lignans	N/A	CUMS	C57BL/6 mice BV2 cells	SPT; FST; OFT	CB2	II	[150]
	*Schisandra chinensis* lignans	N/A	CUMS	C57BL/6 mice BV2 cells, PC12 cells	SPT; FST; OFT	CB2	II	[151]
	*Schisandra chinensis* lignans	N/A	CUMS	C57BL/6 mice BV2 cells	SPT; FST; OFT	CB2	II	[152]
	*Schisandra chinensis* lignans	N/A	CUMS	C57BL/6 mice	SPT; FST; OFT	CB2	II	[153]

Note: Table 1 summarizes representative natural products targeting depression-related GPCRs, including information on experimental models, behavioral evaluations, GPCR involvement, and the current level of supporting mechanistic evidence from preclinical studies, with emphasis on in vivo evidence where available. Clinical evidence and translational considerations are discussed separately in the corresponding sections of the manuscript. Evidence levels: I, direct receptor-target validation; II, pharmacological validation; III, indirect mechanistic associations. When multiple evidence types are available, the highest level of evidence is assigned. Abbreviations: 5-MeO-DMT, 5-Methoxy-N,N-dimethyltryptamine; 5-OH-DMT, 5-Hydroxy-N,N-dimethyltryptamine; BCG, Bacillus Calmette–Guérin; BWT, beam walking test; CHO, Chinese hamster ovary; CORT, cortisol; CRS, chronic restraint stress; CUMS, chronic unpredictable mild stress; EPM, elevated plus maze; FRL, Flinders Resistant Line; FSL, Flinders Sensitive Line; FS, foot shocks; FST, forced swimming test; FUST, female urine-sniffing test; HTR, head-twitch response; HWT, horizontal wire test; LH, learned helplessness; LMA, locomotor activity; LPS, lipopolysaccharide; MCAO, middle cerebral artery occlusion; MI, myocardial infarction; MWM, Morris water maze test; NOR, novel object recognition; NSFT, novelty suppressed feeding test; OFT, open field test; POE, predator odor exposure; SIS, social instability stress; SRS, spatial restraint stress; ST, sucrose splash test; T1DM, type 1 diabetes mellitus; TST, tail suspension test.

## Data Availability

No new data were created or analyzed in this study. Data sharing is not applicable to this article.

## Abbreviations

The following abbreviations are used in this manuscript:

5-HT	5-Hydroxytryptamine/serotonin
5-MeO-DMT	5-Methoxy-N,N-dimethyltryptamine
5-OH-DMT	5-Hydroxy-N,N-dimethyltryptamine/bufotenine
AC	Adenylyl cyclase
AEA	Anandamide
Akt/PKB	Protein kinase B
AMPA	α-Amino-3-hydroxy-5-methyl-4-isoxazolepropionic acid
ATR	Atractylenolide I
BCG	Bacillus Calmette–Guérin
BDNF	Brain-derived neurotrophic factor
CaMKII	Ca^2+^/calmodulin-dependent protein kinase II
cAMP	Cyclic adenosine monophosphate
CAPON	Carboxy-terminal PDZ ligand of neuronal nitric oxide synthase
CB1	Cannabinoid receptor 1
CB2	Cannabinoid receptor 2
CBD	Cannabidiol
cGMP	Cyclic guanosine monophosphate
CHO	Chinese hamster ovary
CORT	Corticosterone
COX-2	Cyclooxygenase-2
CREB	cAMP response element-binding protein
CRH	Corticotropin-releasing hormone
CRS	Chronic restraint stress
CRT	Calreticulin
CUMS	Chronic unpredictable mild stress
DA	Dopamine
DAG	Diacylglycerol
DOP	δ-Opioid
DYN A	Dynorphin A
EA	Echinocystic acid
EC50	Half-maximal effective concentration
ECL	Extracellular loop
ERK	Extracellular signal-regulated kinase
FAAH	Fatty acid amide hydrolase
FSL	Flinders Sensitive Line
FST	Forced swimming test
GABA	γ-Aminobutyric acid
GABAB	γ-Aminobutyric acid type B
GC	Guanylyl cyclase
GDP	Guanosine diphosphate
GFS	Ginseng fruit saponins
GPCR	G protein-coupled receptor
GRb1	Ginsenoside Rb1
GRK	G protein-coupled receptor kinase
GTP	Guanosine triphosphate
HLA	Horizontal locomotor activity
HPA	Hypothalamic–pituitary–adrenal
HSP	Hesperidin
HTR	Head-twitch response
ICL	Intracellular loop
IDO1	Indoleamine 2,3-dioxygenase 1
IL-1β	Interleukin-1β
IL-6	Interleukin-6
IP3	Inositol 1,4,5-trisphosphate
KOP	κ-Opioid
LHb	Lateral habenula
LPS	Lipopolysaccharide
LTD	Long-term depression
LTP	Long-term potentiation
MAO-A	Monoamine oxidase A
MAPK	Mitogen-activated protein kinase
mAChR	Muscarinic acetylcholine receptor
MDD	Major depressive disorder
mEPSC	Miniature excitatory postsynaptic current
mGlu	Metabotropic glutamate
MI	Myocardial infarction
MOP	μ-Opioid
mPFC	Medial prefrontal cortex
mTORC1	Mammalian target of rapamycin complex 1
NAc	Nucleus accumbens
NDRI	Norepinephrine–dopamine reuptake inhibitor
NE	Norepinephrine
NF-κB	Nuclear factor kappa B
NMDA	N-Methyl-D-aspartate
NMDAR	N-Methyl-D-aspartate receptor
nNOS	Neuronal nitric oxide synthase
NAM	Negative allosteric modulator
PFC	Prefrontal cortex
PGC-1α	Peroxisome proliferator-activated receptor gamma coactivator-1α
PIP2	Phosphatidylinositol-4,5-bisphosphate
PKA	Protein kinase A
PKC	Protein kinase C
PLA2	Phospholipase A2
PLC	Phospholipase C
PPAR-γ	Peroxisome proliferator-activated receptor-γ
PSD	Post-stroke depression
PSD-95	Postsynaptic density protein 95
RhoGEF	Rho guanine nucleotide exchange factor
ROCK	Rho-associated coiled-coil-containing kinase
SCL	*Schisandra chinensis* lignans
SERT	Serotonin transporter
SNRI	Serotonin–norepinephrine reuptake inhibitor
SSRI	Selective serotonin reuptake inhibitor
STAT6	Signal transducer and activator of transcription 6
SST	Somatostatin
SAR	Structure–activity relationship
TA	Tannic acid
TCA	Tricyclic antidepressant
TLR4	Toll-like receptor 4
TM	Transmembrane helix
TNF-α	Tumor necrosis factor-α
TPH2	Tryptophan hydroxylase 2
TST	Tail suspension test
T1DM	Type 1 diabetes mellitus
VGLUT1	Vesicular glutamate transporter 1
vmPFC	Ventromedial prefrontal cortex
VTA	Ventral tegmental area
